# EHMTI-0170. The pain is less severe but more disabling in chronic vs. Frequent migraine patients

**DOI:** 10.1186/1129-2377-15-S1-D5

**Published:** 2014-09-18

**Authors:** C Chicu-Hadirca, S Odobescu, L Rotaru, O Grosu, G Corcea, I Moldovanu

**Affiliations:** 1 Neurology, Institute of Neurology and Neurosurgery, Chisinau, Moldova

## Background

Attenuation of some "migrainous" traits during migraine chronification has previously been reported, but the detailed analysis of a migrenous attack structure on a big sample of chronic migraine (CM) in comparison with frequent migraine (FM) patients has not been performed.

## The aim

To evaluate the phenomenology of migraine attack and related phenomena in CM vs. FM.

## Methods

The study included 154 patients with CM and 70 with FM diagnosed accordingly the ICHD-II(2004) and ICHD-IIR (2006) criteria. Detailed clinical features of migraine attacks were analyzed through a complex structured questionnaire.

## Results

Paroxysmal headache in CM is less intensethan in FM (6.7 vs. 7.9 on VAS), significantly rarer hemicranic(35.1% vs. 52.9%) and pulsating (68.2% vs. 85.7%) than FM. Headache in occipito-cervical location is more common in CM(27.2% vs.14.3% FM,p<0.05), correlating with cervical myofascial syndrome (47.4% vs. 11.4%, respectively, p<0.0001). Nausea (63.6% vs. 81.4%, p<0.01) and vomiting (20.8%vs. 30.0%) are less pronounced in CM vs. FM. Associated signs like photophobia (85.7% vs. 97.1%), phonophobia (81.2% vs. 94.3%), osmophobia (71.4% vs. 87.1 %) are also significantly lower in CMvs. FM. In contrast, in CMthe need for bed rest,dizziness, weakness, drowsiness, anxiety, concentration difficulties and decreased work capacity were significantly more frequent during an attack.

## Conclusions

On a big sample size, we have revealed “blurred” migrainous features in CM patients: the attacks "lose" some of pulsatility and hemicranic character, become almost daily, less intense, but more incapacitating and the patient’s suffering is more pronounced.

No conflict of interest.

